# Ultrasound-targeted microbubble destruction remodels tumour microenvironment to improve immunotherapeutic effect

**DOI:** 10.1038/s41416-022-02076-y

**Published:** 2022-12-03

**Authors:** Senbo Liu, Yan Zhang, Yang Liu, Wenkang Wang, Shuochen Gao, Weitang Yuan, Zhenqiang Sun, Lin Liu, Chengzeng Wang

**Affiliations:** 1 Department of Colorectal Surgery, The First Affiliated Hospital of Zhengzhou University, 450052 Zhengzhou, Henan China; 2 Henan Institute of Interconnected Intelligent Health Management, The First Affiliated Hospital of Zhengzhou University, 450052 Zhengzhou, Henan China; 3 Department of Ultrasound, The First Affiliated Hospital of Zhengzhou University, 450052 Zhengzhou, Henan China; 4 Department of Radiotherapy, Affiliated Cancer Hospital of Zhengzhou University, Henan Cancer Hospital, 450008 Zhengzhou, China; 5 Department of Breast Surgery, The First Affiliated Hospital of Zhengzhou University, 450052 Zhengzhou, Henan China

**Keywords:** Cancer microenvironment, Tumour immunology, Cancer immunotherapy

## Abstract

Cancer immunotherapy (CIT) has gained increasing attention and made promising progress in recent years, especially immune checkpoint inhibitors such as antibodies blocking programmed cell death 1/programmed cell death ligand 1 (PD-1/PD-L1) and cytotoxic T lymphocyte-associated protein 4 (CTLA-4). However, its therapeutic efficacy is only 10–30% in solid tumours and treatment sensitivity needs to be improved. The complex tissue environment in which cancers originate is known as the tumour microenvironment (TME) and the complicated and dynamic TME is correlated with the efficacy of immunotherapy. Ultrasound-targeted microbubble destruction (UTMD) is an emerging technology that integrates diagnosis and therapy, which has garnered much traction due to non-invasive, targeted drug delivery and gene transfection characteristics. UTMD has also been studied to remodel TME and improve the efficacy of CIT. In this review, we analyse the effects of UTMD on various components of TME, including CD8^+^ T cells, tumour-infiltrating myeloid cells, regulatory T cells, natural killer cells and tumour vasculature. Moreover, UTMD enhances the permeability of the blood-brain barrier to facilitate drug delivery, thus improving CIT efficacy in vivo animal experiments. Based on this, we highlight the potential of immunotherapy against various cancer species and the clinical application prospects of UTMD.

## Background

Cancer immunotherapy (CIT) is one of the standard therapies along with surgery, chemotherapy, radiotherapy and targeted therapy for cancer treatment, which produces antitumor effects by activating the body’s immune system [1]. Among oncology drug treatments, it is the fourth therapy after chemotherapy, targeted therapy, and hormone therapy. In recent years, immunotherapy, especially with immune checkpoints as therapeutic targets, has made an enormous difference for terminal cancer patients [2]. However, objective tumour response rates are still poor and treatment sensitivity needs to be further promoted [3]. The tumour microenvironment (TME) is the surroundings on which tumour cells rely, and the characteristics of TME significantly affect cancer progression and metastasis, correlating with the efficacy of CIT [4]. As tumour cells proliferate, the TME is dynamically changing, and the negative regulatory mechanisms of the immune system are used by TME to counteract the antitumor immune response and ultimately promote tumour immune escape [5]. However, one of the biggest challenges of current CIT is the narrow range of patients and the remarkable individual differences, which may be attributed to the heterogeneity of the TME [6, 7].

Traditionally, ultrasound is an imaging technique for diagnosis, but in the last few years, it has been developed for therapeutic purposes [8], one of which is ultrasound-targeted microbubble destruction (UTMD). UTMD has gained widespread popularity with regard to its non-invasive, targeted drug delivery and gene transfection characteristics [9]. Numerous studies on the application of UTMD to cancer immunotherapy have revealed the extraordinary possibility of remodelling TME and enhancing CIT efficacy [10–12], which has the potential to break down the heterogeneity of the TME and reach more patients.

In this review, we summarise the influences of UTMD on various components of TME to indicate its role in remodelling the TME. Meanwhile, the delivery role of this technology in vivo and its potentiation of CIT will also be discussed in this paper.

## Ultrasound-targeted microbubble destruction

UTMD is an ultrasound therapy technique that uses low-frequency ultrasound to stimulate the cavitation of microbubbles in vivo, resulting in oscillation (alternating contraction and expansion) and rupture [13]. This process generates mechanical energy such as fluid flow, shear stress, shock wave and microjet, causing the cavitation effect or sonoporation [14, 15]. Unlike high-intensity focused ultrasound (HIFU), the low ultrasonic frequency of UTMD ranges from 20 kHz to 1 MHz, and its thermal effect is fragile [16]. The introduction of microbubbles (exogenous cavitation nuclei) can increase the density of cavitation nuclei, lower the cavitation threshold and improve the safety of ultrasound [17].

Current researches conclude that the biophysical mechanism of UTMD is mainly the cavitation effect (or called sonoporation). The cavitation effect refers to the vibration of vapour or gas cavities under the action of acoustic waves. Then vapour or gas microbubbles expand, collapse and rupture when the acoustic pressure reaches a particular threshold [18]. The effect includes two forms. One is stable cavitation, where the microbubbles alternately expand and contract with changes in acoustic pressure, producing a microfluidic jet with low shear [19–21]. The other is inertial cavitation, where the microbubbles overextend and rupture to build a large shear [20, 21]. The cavitation effect induces the increase in the gap between vascular endothelial cells and the permeability of cell membrane [22, 23]. What’s more, microbubbles are an excellent delivery vehicle for targeted and specific delivery of bioactive molecules (such as drugs, nanoparticles, vaccines and therapeutic genes) to organs or tissues within ultrasound reach [24–26]. Microbubbles not only protect biologically active molecules from endogenous clearance during transport, but also release them in the focal area through inertial cavitation, enabling targeted therapies and reducing systemic toxic side effects [24, 27].

## Tumour microenvironment and cancer immunotherapy

The TME is the complex environment where tumour cells or cancer stem cells grow. The comprehensive system consists of tumour cells, immunocytes, mesenchymal cells, tumour-associated endothelial cells (TAECs), extracellular matrix (ECM), cytokines, and other molecules that contribute to tumour growth and progression, such as immune checkpoint [28–30]. CIT is one of the most promising cancer treatment strategies, including bacterial immunotherapy, cytokine therapy, monoclonal antibody, immune checkpoint inhibitors (ICIs), cancer vaccines, adoptive cell therapy (ACT) and oncolytic virus therapy (OVT) [31–33]. These therapies can augment the recruitment of T cells in the TME, block the binding of immune checkpoints, enhance exposure to antigens or directly lyse tumour cells [33–36]. Although CIT has been successfully applied to many types of malignant tumours, only a small percentage of strictly selected advanced cancer patients benefit from these therapies [37].

The complicated and dynamic TME can influence the efficacy of immunotherapy. First, the TME contains immunosuppressive cells and a large number of cytokines such as regulatory T cells (Tregs), myeloid-derived suppressor cells (MDSCs), M2 type macrophages, IL-1, TGF-β, which retard the activation of effector cells especially T cells [38, 39]. Second, tumour cells reduce surface expression of HLA-I, making it difficult to be processed for presentation and not recognised by the immune system [40]. Third, tumour antigens are not adequately delivered to the lymphoid tissue due to the obstruction of the TME, predicting inefficient activation of T cells [41]. Moreover, the hypoxic and acidic microenvironment causes deactivation, senescence and depletion of infiltrating T cells [42].

In brief, the TME is the critical factor in influencing tumour immune response. The efficiency and sensitivity of CIT are closely correlated with the TME. An in-depth understanding of the interplay between the TME and immunotherapy is significant.

## Effects of UTMD on intratumoral CD8^+^ T cells

CD8^+^ T cells play an essential role in tumour-mediated immune responses by recognising specific tumour antigens. Tumour cell necrosis induced by the perforin–granzyme pathway and apoptosis induced by the FAS pathway are two important antitumor mechanisms of CD8^+^ T cells [43]. Moreover, IFN-γ secreted by CD8^+^ T cells can cause tumour cell ferroptosis, an iron-dependent and non-apoptotic form of cell death [44]. However, CD8^+^ T cells are not sufficiently infiltrated and cannot be fully activated in most solid tumours [19]. There are many factors that impede CD8^+^ T-cell infiltration and activation during tumour progression, such as reduced tumour perfusion [45], blocked antigen presentation [40], high-potassium environment [46] and high expression of inhibitory receptors [47]. Appropriate ways to promote CD8^+^ T-cell infiltration and activation are significant.

**Fig. 1:**
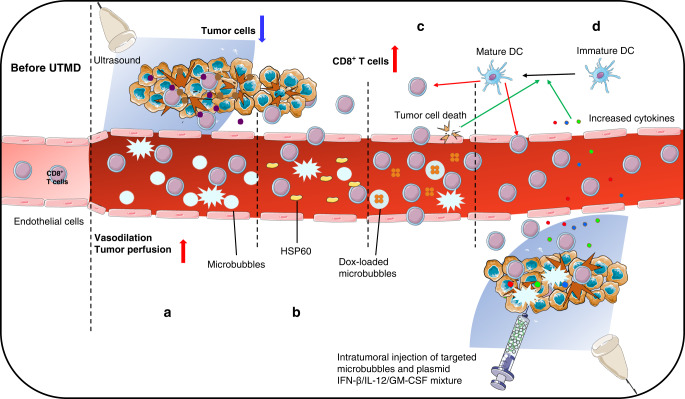
UTMD increases the infiltration of CD8^+^ T cells. **a** Vasodilation and enhanced tumour perfusion. **b** HSP60 attracts infiltration of CD8^+^ T cells. **c** and **d** Dox-mediated tumour cell death and increased cytokines promote the maturation of DCs to cause CD8^+^ T-cell infiltration.

One recent study showed that the UTMD treatment increased the percentage of infiltrating CD8^+^ T cells by 24.28%, and the absolute number was higher with a low mechanical index (MI = 0.4). Furthermore, the combination therapy of UTMD with anti-programmed cell death ligand 1 (anti-PD-L1) boosted the secretion of IFN-γ and granzyme B, indicating the activation and function of infiltrating CD8^+^ T cells. Vasodilation and enhanced tumour perfusion contributed to this phenomenon because the suppressive tumour immune response was mitigated by improving tumour hypoxia [48] (Fig. 1a). In addition, mechanical stress by UTMD promotes heat-shock protein (HSP60) overexpression, thereby increasing the infiltration of CD8^+^ T cells into the tumour [49] (Fig. 1b). The underlying mechanism may be that HSP binding to scavenger receptor SREC-I or LOX-1 (HSP-specific receptors expressed on dendritic cells) activates dendritic cells and mediates the cross-presentation of antigenic peptides from tumour cells, thereby triggering the T-cell-mediated adaptive immune responses [50]. Other studies also found similar results by combining microbubbles with plasmids encoding cytokines, such as pIFN-β and pIL-12 [51, 52] (Fig. 1d). What’s more, UTMD was reported to enhance Dox-mediated immunogenic cell death (ICD) to activate more dendritic cells (DCs), eliciting an increment in the percentage of CD8^+^ T cells [53] (Fig. 1c). Similarly, UTMD with GM-CSF plasmids was shown to conduct recruitment of CD8^+^ T cells by upregulating DCs maturation [54] (Fig. 1d). Inertial cavitation mediated by UTMD exposes intracellular nucleic acids, pathogen-associated molecular patterns (PAMP), or damage-associated molecular patterns (DAMP) [55]. Cytosolic double-stranded DNA (dsDNA) and dsRNA are detected by cyclic GMP-AMP synthase (cGAS) and retinoic acid inducible gene I (RIG-I), which are pattern-recognition receptors (PRRs), resulting in the activation of stimulator of interferon genes (STING) [56]. The ubiquitinated STING elicits the expression of pro-inflammatory factors through IRF3 and NFκB-dependent pathways. Meanwhile, activated STING in antigen-presenting cells stimulates the T-cell-mediated adaptive immune responses and exerts anti-tumour effects [57]. In consequence, UTMD enhances CD8^+^ T-cell infiltration and T-cell-mediated adaptive immune responses by dilating blood vessels, promoting HSP expression, boosting tumour antigen exposure, and facilitating cytokine secretion.

## Effects of UTMD on tumour-infiltrating myeloid cells

**Fig. 2:**
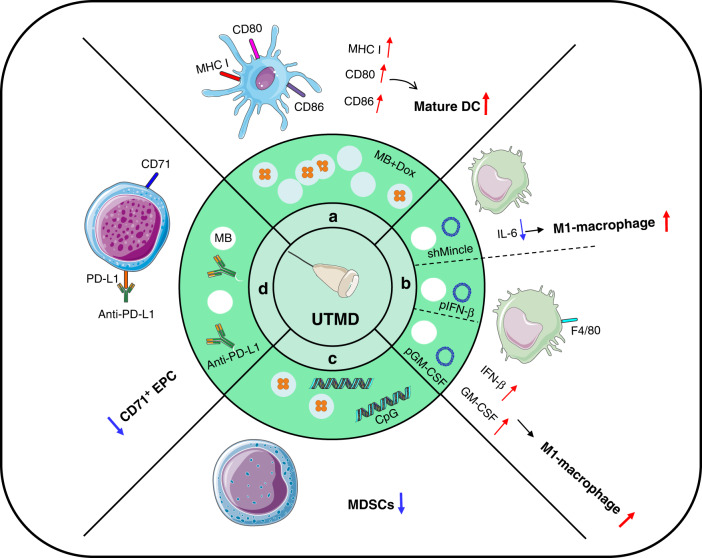
Effects of UTMD on tumour-infiltrating myeloid cells. **a** US + MB + Dox treatment promotes the maturation of DCs. **b** US + MB + shMincle/pIFN-β/GM-CSF treatment increases M1-macrophage infiltration. **c** US + MB + Dox+CpG treatment reduces the frequency and absolute amount of MDSCs. **d** US + MB + anti-PD-L1 treatment decreases the frequency of CD71^+^ erythroid progenitor cells.

Tumour-infiltrating myeloid cells (TIMs) are a non-negligible regulatory component in tumour progression, including mast cells, DCs, monocytes, and macrophages [58, 59]. DCs are a specialised class of antigen-presenting cells (APCs) derived from the hematopoietic bone marrow [60]. Tumour-infiltrating DCs process and present tumour antigens and gradually differentiate into mature DCs that express co-stimulatory molecules CD80 or CD86 to interact with T cells to resist tumours [61]. UTMD was explored to heighten antigen processing via MHC class I to increase CD86 expression in the meninges, indicating the maturity of DCs. UTMD alone or combined with Dox mediated the release of DAMPs (such as HMGB-1, HSP, ATP) from tumour cells [53, 62]. These molecules bind to pattern-recognition receptors (such as Toll-like receptors, and purinergic receptors) to stimulate DCs maturation [63]. Upon DCs maturation, an increased expression of TAP1 and TAP2 was detected, which promotes the loading of MHC-I [62]. In addition to the enriched CD86, other studies confirmed the maturation and activation of DCs because of the enhanced CD80 expression and INF-γ (active marker) secretion by UTMD [64] (Fig. 2a).

Tumour-associated macrophages (TAMs) and MDSCs play an immunosuppressive role in the TME to promote malignant tumour progression [65, 66]. TAMs are generally divided into M1 and M2 phenotypes and are interchangeable between the two, with the former being antitumor and the latter pro-tumour [67]. Xue et al. bound shMincle to microbubbles and then applied UTMD to deliver the complexes to the tumour region. Ultimately, they found that UTMD blocked the Mincle/Syk/NF-κB axis by targeting Mincle and, more excitingly, promoted the conversion of the M2 phenotype to the M1 phenotype [68] (Fig. 2b). Complementarily, an increase in the number of F4/80^+^ macrophages infiltrates was also observed by combining plasmids with microbubbles [51, 54] (Fig. 2b). Macrophages have been reported to stimulate the expression of pro-inflammatory factors via the TLR4-MyD88 pathway [69]. Accordingly, we speculate that DAMPs generated by the cavitation effect are recognised by TLRs and then activate the downstream signalling molecule interferon regulatory factor (IRF)-5 via the TLR4-MyD88 pathway [70]. IRF-5 phosphorylates and enters the nucleus to perform transcriptional functions, thus promoting macrophage polarisation to the M1 phenotype [71]. Moreover, Kheirolomoom established a bilateral metastatic breast cancer model in which one side was treated with UTMD combined immune adjuvant CpG and the other was left untreated. CpG as an agonist of TLR9 has an essential supporting role in reversing the suppressive TME [72]. Within the both sides, there was a substantial drop in the frequency and absolute amount of MDSCs after performing the treatment procedure [73] (Fig. 2c). Similarly, one recent study elucidated that UTMD decreased the frequency of CD71^+^ erythroid progenitor cells, which suppress the immune response [74] (Fig. 2d). In conclusion, UTMD facilitates the expression of co-stimulatory molecules, triggers the maturation of DCs, promotes the polarisation to pro-inflammatory macrophages and curbs MDSCs to regulate the immune-suppressive TME.

## Effects of UTMD on intratumoral regulatory T cells

Tregs are a group of immunosuppressive cells that maintain immune homoeostasis in the body. Tregs inhibit antitumor immune responses and promote tumour progression [75]. The chemokine receptor CCR4 expressed by Tregs selectively binds to the chemokine ligand CCL22, which is highly expressed in most tumour tissues [76, 77]. The immune response is downregulated mainly in two ways. First, Tregs directly inhibit effector cells through releasing perforin, granzyme or expressing inhibitory receptors programmed cell death protein 1 (PD-1) and cytotoxic T lymphocyte-associated antigen 4 (CTLA-4) or secreting inhibitory cytokines such as TGF-β, IL-10 and IL-35 [78, 79]. Second, Tregs indirectly suppress T-cell activation by interacting with APCs. CTLA-4 expressed on Tregs conjugates with the co-stimulatory molecule CD80/86 on APCs. This process impedes the binding of CD28 on T cells to CD80/86, thereby inhibiting T-cell activation [80].

UTMD treatment could reduce Tregs in the tumour [48], and the proliferation rate of Tregs gradually decreases with increasing ultrasound irradiation time due to apoptosis [81]. Interestingly, the combination of UTMD and doxorubicin (Dox) was able to induce the translocation of calreticulin (CRT) and ER-associated protein disulfide isomerase ERp57 and upregulate the expression of chromatin-binding protein high-mobility group B1 (HMGB-1) in tumour cells, suggesting the activation of ICD. This response reversed the suppressive immune microenvironment so that a lower percentage of Tregs was observed [53]. Pro-inflammatory factors have been reported to differentiate Tregs into an unstable subpopulation. This subpopulation decreased the expression of Forkhead family transcription factor P3 (Foxp3) and Treg-related markers, raised the expression of TNF-α and IL17A, and elevated both glycolysis and proliferation [82]. UTMD boosts the expression of pro-inflammatory factors and may deregulate the immunosuppressive function of partially Tregs in this manner. Additionally, concomitant UTMD and microRNA was also used to knock down Foxp3 to inhibit the negative effect of Tregs by decreasing the level of TGF-β and IL-10 [83]. Mature Tregs maintain sustained Foxp3 transcription through the autoregulatory transcriptional circuit [84]. Once Foxp3 is knocked down, this circuit is disrupted and Tregs lose immunosuppressive function. Thus, UTMD combined with drugs or microRNAs dysfunction the proliferation and maturation of Foxp3^+^ Tregs, thereby alleviating the unfavourable tumour microenvironment.

## Effects of UTMD on intratumoral natural killer cells

Natural killer cells (NK cells) as critical components of the innate immune response are a class of lymphocytes that lyse tumour cells and infected cells non-specifically without pre-sensitisation [85]. NK cells recognise tumour cells through two modes: missing-self and antibody-dependent cell-mediated cytotoxicity (ADCC), depending on the balance between activatory and inhibitory receptors [86–88].

Considering the non-antigen-specific killing mechanism, NK cells may not only resist tumour escape caused by antigenic drift [89], but also have the potential to exert broad antitumor effects on a wide range of tumour species [90]. However, suppressed activity immensely limits the advancement of NK cells’ clinical applications. Alkins et al. injected HER2-specific NK-92 cells into tumour-bearing mice via tail vein. Then they found a 5-fold increase in the ratio of NK-92 cells to tumour cells compared to the control group with ultrasonication performed 30 seconds after injection. However, the detailed mechanism leading to increased NK cell infiltration is not precise. One possibility is that ultrasound transiently enlarges the endothelial cell gap to facilitate the passage of NK cells [91]. What’s more, pro-inflammatory chemokines recruit circulating NK cells to tumour sites [87]. UTMD could trigger the expression of chemokine (C-X3-C motif) ligand 1 (CX3CL1), thus attracting NK-92MI cells into the tumour regions [92]. The mechanical stimulation generated by cavitation promoted Ca^2+^ influx through the mechanosensitive channel Piezo1 [93]. In the presence of Ca^2+^, the perforin released from NK cells is embedded in the tumour cell membrane [94], thus killing tumour cells. Therefore, UTMD expands the endothelial cell gap, elicits pro-inflammatory chemokine expression, and promotes Ca^2+^ influx to potentiate intratumoral NK cell recruitment and lytic effects.

## Effects of UTMD on tumour vasculature

Tumour vasculature is tortuous, tangled, branched irregularly and unevenly distributed, with low pericyte coverage and loose connections between endothelial cells, which augments interstitial fluid pressure (IFP) [95]. Blood does not flow in a steady, unidirectional course through tumours, and high interstitial pressure collapses blood vessels and reduces tumour perfusion [96]. Furthermore, as the main cytokine, VEGF effectively stimulates the pathological process, forming abnormal tumour blood vessels [97, 98]. VEGF has also been utilised as a target to normalise tumour vasculature [99]. Normalisation of tumour vasculature improves the supply of partial nutrients and oxygen, leading to a return of tumour sensitivity to drugs [100]. In addition, this modifies the acidic and hypoxic hostile environment and diminishes the number of immunosuppressive cells [101]. On the other hand, normalisation alleviates high IFP in the tumour, facilitating the delivery of ICBs and infiltration of effector immune cells, thus improving the efficacy of CIT [99].

UTMD significantly increased perfusion according to ultrasound imaging and decreased tumour microvascular density (MVD) [48], demonstrating vascular normalisation. Previous studies have revealed that ultrasound using microbubble cavitation increased shear-dependent ATP. ATP release mediated calcium wave propagation to activate calcium-dependent purinergic signalling pathways. Endothelial nitric oxide synthase and vasoactive prostanoids were produced to augment skeletal muscle blood flow [102]. The expression of CD34 (positioned on tumour vascular endothelium), VEGF and tumour endothelial marker (TEM8) decreased, suggesting the inhibition of tumour angiogenesis [103, 104]. Consistently, histological staining revealed vasodilation rather than angiogenesis mediated by UTMD [48, 105]. Besides, Liu reported that UTMD allowed FITC-labelled dextran to leak from the vasculature into the extravascular tumour area, and no increase in apoptotic cells was distinctly observed [49]. Tumour vascular permeability was better enhanced without significant cellular damage, facilitating homogeneous drug distribution in the tumour’s central and peripheric regions [49, 106, 107]. What’s more, UTMD promotes the expression of the adhesion molecule ICAM-1, which is crucial for T-cell accumulation [48]. Tumour vascular normalisation has been reported to upregulate the expression of adhesion molecules and thus facilitate the localisation of antitumor T cells [108]. UTMD potentially increases the accumulation of T cells by contributing to the normalisation of tumour vasculature. Taken together, UTMD fosters vasodilation, strengthens vascular permeability, restrains tumour angiogenesis, and normalises tumour vasculature, thereby accumulating and seeding antitumor effector immune cells.

## Effects of UTMD on barrier structures

The blood-brain barrier (BBB) strictly limits the entry and exit of substances to protect the brain from toxic molecules [109]. When brain tumours occur (both primary and secondary brain tumours), components of the BBB will be modified to develop the blood-tumour barrier (BTB) [110], such as increased VEGF expression, loss of tight junctions and pericytes [111], and loss of basement membrane protein components [112]. Although some drugs can enter the tumour through BTB, the therapeutic effect is still unsatisfactory [110].

Recent studies demonstrated that UTMD was a technology worth exploring for opening BBB/BTB. For instance, researchers found a temporary increase in BBB permeability by UTMD comparing MRI signal intensity change (SIC) at different time points, including a significant increase in CD4^+^ and CD8^+^ lymphocytes in the rat glioma model [113]. In addition, in intracranial melanomas, UTMD successfully disturbed BTB, enhanced the expression of the pro-inflammatory cytokines and enriched the inflammatory gene sets. More importantly, UTMD elicited immune response due to the raised expression of H2-K1 and H2-D1, the class I MHC molecules [62]. In brain metastases, some investigators showed that UTMD increased the extravascular concentration and penetration distance of chemotherapeutic agents, suggesting the possibility of improved efficacy [114]. Another modified critical component is the transporter, Permeability-glycoprotein (Pgp), which overexpresses on the BBB/BTB. Aryal reported that using UTMD could transiently downregulate Pgp expression, supported by being suppressed for 48 hours and restored after 72 hours at the ultrasound intensity of 0.55MPa [115]. Similarly, UTMD significantly increased paclitaxel-induced apoptosis by strengthening the permeability of the blood-prostate barrier [116].

## Enhanced antitumor efficacy in different cancer species with UTMD

**Fig. 3:**
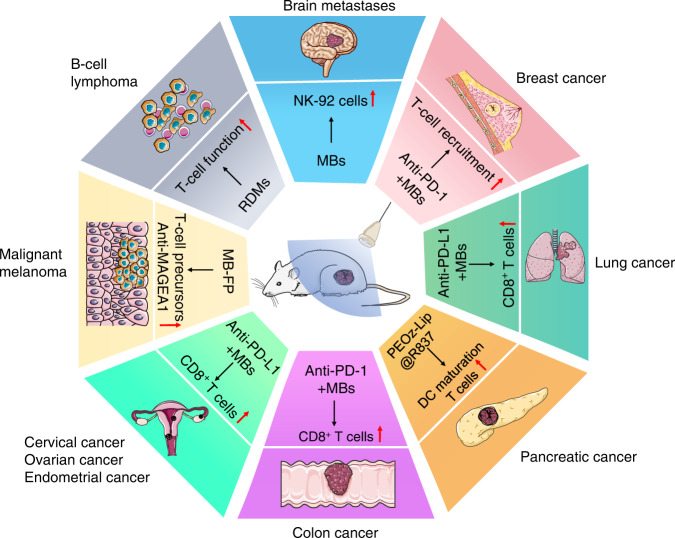
UTMD strengthens antitumor efficacy in different cancer species. UTMD has been tested in animals and achieved enhanced antitumor effects in a variety of cancers by augmenting immune responses.

As previously mentioned, UTMD can increase vascular permeability, normalise tumour vasculature, increase infiltration of antitumor effector cells and reduce immunosuppressive cells in the TME. This technology has led to investigating its antitumor efficacy and promising results have been achieved (Fig. 3).

UTMD reduced mean brain metastasis volumes by 3-fold (measured at 28 days) and prolonged the mean survival time by 3-fold of rats with the front-loaded treatment arm (5 treatments in the first week, 2 treatments in the second week, and 1 treatment in the third week) by injecting Definity microbubbles, which was attributed to the localisation of NK-92 cells within the tumour [10]. UTMD with pIFN-β and anti-PD-1 also slowed tumour growth by 4-fold and even showed complete tumour regression by enhancing T-cell recruitment in a mouse model of breast cancer [51]. For the Lewis lung cancer (LLC) model, UTMD and anti-PD-L1 combination therapy induced an increase in IFN-γ production, causing an increase in CD8^+^ T-cell frequency to inhibit LLC progression [74]. Moreover, researchers constructed TLR agonist (R837)-loaded pH-responsive liposomes (PEOz-Lip@R837) to treat pancreatic cancer. They observed a 1.5-fold lower mean fluorescence intensity of primary tumour, a 5-fold smaller mean distant tumour volume compared to the control group and reduced secondary tumour weight value due to DC maturation, and activation and proliferation of T cells after UTMD [117].

Colon cancer was also treated using UTMD technology with anti-PD-1, and the number and effector function of CD8^+^ T cells were systemically increased, thereby augmenting anti-PD-1 efficacy [118]. For gynaecologic tumours, UTMD combined with bevacizumab or plasmid pCMV-IL-12 or anti-PD-L1 antibody enhanced efficacy in cervical, endometrial, and ovarian cancers by increasing the migration and infiltration of CD8^+^ T cells [52, 119, 120]. In addition, UTMD has also been studied for the therapy of malignant melanoma. Gao et al. reported that UTMD with MB-FP (gas-filled ultrasound microbubbles with HSP70-melanoma associated antigen A1 fusion protein) stimulated the generation of T-cell precursors and boosted the titre of anti-MAGEA1, indicating both boosted cellular and humoural immune responses [121]. Another study on the treatment of B-cell lymphoma found that rituximab-conjugated and Dox-loaded microbubbles (RDMs) significantly reduced tumour proliferation, which two out of five mice achieved tumour-free, and promoted apoptosis of malignant cells through restoring T-cell function, suggesting effective antitumor immune responses [122].

## UTMD in delivery

**Fig. 4:**
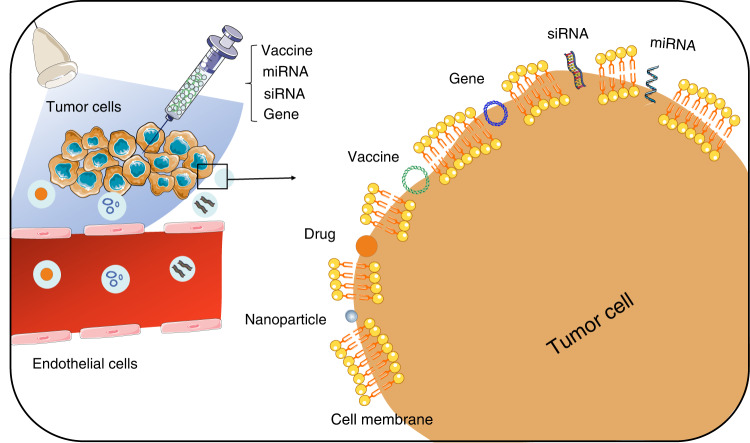
UTMD for delivery applications. UTMD improves the efficiency of drugs, nanoparticles, vaccine delivery and gene, siRNA and miRNA transfection.

UTMD reversibly opens tight junctions between cells and temporarily expands the endothelial cell gap, providing accessible pathways for drugs, genes, plasmids, and nanoparticles [25] (Fig. 4). It is a non-invasive and efficient delivery process. Therefore, it may be a novel and promising delivery technique that combines microbubbles with relevant effector substances and then delivers them to the target location.

### Drugs

Drugs are widely utilised in the preoperative and postoperative treatment of malignant tumours and have achieved certain efficacy. However, the urgent problem is that drugs penetrate weakly into the tumour, resulting in a relatively high concentration when achieving the expected antitumor effect, which may cause severe toxic side effects. UTMD has been conducted to study to improve this situation. The sonoporation not only normalises tumour vasculature in TME, but also opens the endothelial cell gap and augments the permeability of the tumour cell membrane, which facilitate the delivery of drugs into the tumour. For example, paclitaxel combined with folate-targeted microbubbles was delivered to ovarian cancer sites. Then the higher drug concentrations were detected in tumour and lymphoid tissues, while lower in blood, implying better efficacy and less severe side effects [123]. Consistently, tumour cells accumulated seven times higher Dox with an exceeding five times penetration distance [53, 114]. In addition, the higher delivery efficiency of anti-PD-L1 was discovered with UTMD treatment by detecting fluorescent signals in a mouse intracranial glioma model [124]. UTMD was also able to improve the consistency of Dox distribution, reducing the difference in concentration between the central and peripheral regions of the tumour [107].

### Cancer vaccines

Cancer vaccines are designed to trigger the specific immune response and establish immune memory to prevent tumour metastasis and recurrence [125]. Cancer vaccines work primarily by processing tumour-associated antigens (TAAs) or tumour-specific antigens (TSAs) through APCs to activate effector cells [126]. DNA and RNA vaccines are also common types. Although APCs can internalise DNA and RNA, the low uptake efficiency limits the clinical effectiveness of these vaccines [127]. Moreover, insufficient intratumoral CD8^+^ T-cell infiltration and local accumulation of immunosuppressive cells such as Tregs, MDSCs, and TAMs are essential factors compromising vaccine efficacy [125]. Given its remodelling of TME, UTMD is a promising novel platform for vaccine delivery to improve cancer vaccine efficacy. Some progress has been made in applying UTMD technology to deliver DNA or RNA vaccines. For instance, antigen mRNA was co-cultured with DCs and then treated with sonication for 30 seconds to obtain mRNA sonoporated DCs. After being injected with tumour cells, the tumour-bearing mice received two inoculations of therapeutic mRNA sonoporated DCs. Eventually, the mice had 58% slower tumour growth, 41% longer median survival and elicited immune memory by reinjection 42 days following the initial tumour injection [128]. What’s more, pGM-CSF-loaded microbubbles with UTMD mediated more cytokine secretion and recruited more T cells on both the treated and untreated sides of the bilateral tumour models [54]. Moreover, pUb-M-loaded bubble lipoplexes (containing DNA encoding melanoma-specific antigens) were constructed to immunise mice. Furthermore, they found that 7/10 of melanoma mice developed complete tumour rejection. Inhibition was observed not in subcutaneous tumours but pulmonary metastases when tumour cells were reinjected 100 days after the initial implantation, suggesting the successful establishment of immune memory [129].

### Molecules

MicroRNA, short interfering RNA, genes and nanoparticles have also made some progress in tumour therapy [130, 131]. miRNAs have been shown to participate in reprogramming the TME, including tumour-infiltrating lymphocytes, MDSCs, cancer-associated endothelial cells, to accelerate or dampen tumour progression [132]. UTMD has the potential to augment the antitumor effect and alleviate the immunosuppressive environment through the delivery of miRNAs. What’s more, traditional viral vectors often raise concerns about their immunogenicity in humans, while physical methods such as electroporation are less specific and with low transfection efficiency [133]. UTMD may be a novel and adequate technology due to its reversibility, non-invasiveness and repeatability. The delivery of miR-34a into cervical cancer cells using UTMD was found to have a stronger fluorescence emission, suggesting a higher gene transfection efficiency. Simultaneously, the binding of PD-L1 Ab to miR-34a lorded microbubbles was observed to cause an increase in apoptosis by detecting the mRNA expression of Bax and Bcl2 [134, 135]. Besides, UTMD was reported to enhance anti-miR21 delivery efficiency by 5-fold through modified nanoparticles. The distribution of the Cy5 fluorescence signal showed that hepatocellular carcinoma cells successfully absorbed miRNAs released by nanoparticle rupture [136]. In addition to miRNA, UTMD is also used to deliver siRNA, indicating a 10-fold increase in siRNA delivery in brain TME [137]. Moreover, researchers constructed a suspension of DNA plasmids and microbubbles to deliver endostatin (ED) and CRT DNA to muscle tissue. They observed a significant increase in ED and CRT expression and a decrease in tumour size combined with Dox in orthotopic hepatocellular carcinoma and lung cancer [138].

## Clinical application potential of UTMD

In the background of the popularity of precision medicine and targeted therapy, UTMD is attracting attention for its unique advantages. It also demonstrates substantial prospects for clinical application. First, UTMD is safe and harmless to the heart, spleen, kidneys and other organs [74, 92]. Studies have shown that UTMD did not cause significant weight loss or a decrease in cellular activity in experimental animals [116, 139]. Second, the technique is repeatable in opening the BBB, and the microbubbles remain well stabilised during the movement in the circulatory system in vivo [91, 140, 141]. Third, UTMD is highly specific and targeted by modifying microbubbles to confer them localisation ability [25, 142]. Furthermore, boosting CIT is one of the most clinically valuable aspects. For instance, compared to using them alone, UTMD strengthened the antitumor effects of ICIs and cytokines, such as anti-PD-1/PD-L1 and IL-12 [143–145]. What’s more, UTMD lessened the toxic effects of chemotherapy drugs by encapsulating them in microbubbles, creating a barrier between them and organs or tissue [107]. More interestingly, this technology could regulate the release rate of the drugs or molecules and precisely kill cells in the tumour area without disturbing the level of immune cells throughout the body, which may alleviate the immune deficiency caused by chemotherapy [68, 146].

## Discussion

UTMD exerts a notable effect on TME by increasing infiltration of CD8^+^ T cells and NK cells, facilitating maturation of DCs and conversion of macrophage phenotype, diminishing the number of Tregs and MDSCs and normalising tumour vasculature, thereby remodelling TME. In addition, UTMD can also break down barrier structures, promote drug delivery, gene transfection, and sensitise cancer vaccines.

While focusing on the substantial promise for clinical application, some limitations and difficulties need to be further addressed. (a) Currently, the majority of UTMD trials are still being conducted in animals. Although a few clinical trials are available, they evaluate whether to improve the sensitivity of chemotherapy and radiotherapy [147–149]. Considering the complexity and heterogeneity of the human environment, the application of UTMD combined with CIT necessitates further clinical trials to discover. (b) Neoplasms at varying regions or organs have either mild or pronounced differences in the TME, which affects the selection of immunotherapy regimens. Accordingly, taking into account the immune heterogeneity of the organs and individual genetic background and then choosing the well-matched microbubbles is a part of the UTMD treatment strategy that cannot be overlooked and merits further investigation. (c) The current commercially available microbubbles are only used for enhanced imaging, and the “next generation” microbubbles integrated with drugs, genes and vaccines need to be optimised and adapted for clinical application in the future. In other words, the new generation microbubbles are not only a complex containing multifunctional substances, but also convenient to produce, preserve and transport so that they can be successfully used in clinical patients. (d) The optimal parameters of UTMD are continuously being investigated. The therapeutic effect is not only correlated with ultrasound parameters such as frequency, mechanical index, duty cycle and irradiation time, but also with non-ultrasound elements such as microbubble dose, ambient temperature, air humidity and tissue type. The appropriate microbubble type and acoustic parameters are pivotal segments of the treatment protocol. (e) Most animal treatments utilise ultrasound to irradiate solid subcutaneous tumours in mice. For the more prominent human body, the suitable ultrasound probe and irradiation modality for treating haematologic and cavernous organ tumours such as colorectal cancer is a topic worth investigating. (f) The precise mechanisms that UTMD remodels the components of TME have not been elucidated. Previous studies have only focused on surface phenomena, so an in-depth analysis of the regulatory agency causing enhanced immune response may provide fresh insights on CIT sensitisation.

Apart from CIT, UTMD has shown potential in augmenting chemotherapy and radiotherapy. Clinical trials revealed that UTMD combined with nab-paclitaxel, gemcitabine or technetium 99 m macroaggregated albumin had a greater prevalence of tumour response, prolonged quality of life, and extended survival [147–149]. In addition, UTMD has been disclosed to treat cardiovascular diseases, such as avoiding thrombosis after mechanical heart valve replacement, preventing cardiomyopathy, and relieving acute cellular rejection after heart transplantation [150]. More interestingly, UTMD augmented proliferation and chondrogenic and osteogenic differentiation, demonstrating activation of mesenchymal stem cells [151]. What’s more, using UTMD better to diagnose is under ongoing study. Conventional ultrasound with contrast imaging primarily focuses on the perfusion status due to the size of microbubbles and the permeability of the blood vessels. UTMD combined with targeted microbubbles breaks through vascular restrictions to bind to markers of extravascular tissues or tumours, thereby facilitating early diagnosis, clinical staging, and disease monitoring [152, 153].

In short, we conclude that UTMD plays an essential part in remodelling the TME and has an augmenting action on CIT. We believe that as the research on UTMD technology continues, the mystery of its mechanism will gradually unravel and bring a new revolution in tumour immunotherapy.

## Supplementary information

Reproducibility checklist
